# The therapeutic effect of splenectomy plus selective pericardial devascularization versus conventional pericardial devascularization on portal hypertension in China: a meta-analysis

**DOI:** 10.18632/oncotarget.23857

**Published:** 2018-01-03

**Authors:** Yajie Zhao, Chengfeng Wang

**Affiliations:** ^1^ Department of Pancreatic and Gastric Surgery, National Cancer Center/Cancer Hospital, Chinese Academy of Medical Sciences and Peking Union Medical College, Beijing 100021, China

**Keywords:** selective esophagogastric devascularization, non-selective esophagogastric devascularization, portal hypertension, meta-analysis

## Abstract

**Background:**

To systematically review perioperative outcomes and postoperative complications between splenectomy plus s-EGDV and n-sEGDV for portal hypertension complicated with thoracic esophageal varices and bleeding by a meta-analysis.

**Method:**

We searched the databases of PubMed, the Cochrane Library, Web of Science, EMBASE, TCGA, Chinese Biomedicine Database from January 2000 to June 2017, and included studies that compared perioperative outcomes and postoperative complications between s-EGDV and n-sEGDV. These included studies were assessed by two independent investigators.

**Results:**

Seven randomized controlled trials (RCTs) and seven non-randomized observational clinical studies (OCS) were included. The s-EGDV was more beneficial than n-sEGDV in reducing the PVF (OR = 4.26; 95% CI, 2.81–5.71; *P* < 0.00001; *I*^2^ = 97% for heterogeneity), portal vein flow (OR = −111.75; 95% CI, −197.13–26.38; *P* = 0.01; *I*^2^ = 90% for heterogeneity), portal hypertensive gastropathy(OR = 0.38; 95% CI, 0.28–0.51; *P* < 0.00001; *I*^2^ = 0% for heterogeneity), hepatic encephalopathy (OR = 0.40; 95% CI, 0.23–0.71; *P* = 0.002; *I*^2^ = 22% for heterogeneity), postoperative re-bleeding (OR = 0.43; 95% CI, 0.29–0.63; *P* < 0.0001; *I*^2^ = 9% for heterogeneity), postoperative mortality (OR = 0.52; 95% CI, 0.32–0.85; *P* = 0.009; *I*^2^ = 0% for heterogeneity) and in increasing hepatic artery flow (OR = 92.53; 95% CI, 9.60–175.46; *P* = 0.03; *I*^2^ = 95% for heterogeneity).

**Conclusion:**

sEGDV offers a more effective surgical approach with fewer complications to treat portal hypertension than n-sEGDV. Upon further detailed analysis of the surgical indications and hemodynamic and postoperative major complications of selective devascularization, sEGDV likely will provide us with a new direction in the choice of surgical approach for portal hypertension.

## INTRODUCTION

In China, cases of hepatitis leading to liver cirrhosis and portal hypertension are on the rise. The typical clinical manifestations of portal hypertension are splenomegaly and hypersplenism, portacaval collateral, and ascites; in some patients, this is further complicated by esophageal varices and bleeding, ultimately resulting in mortality [1]. Portosystemic shunt (PSS) and gastroesophageal devascularization (GD) are the main surgical treatment methods [2]. PSS operation can significantly reduce portal venous pressure, and the rate of bleeding can be controlled by 85%–100%. Further, PSS can not only eliminate ascites in a rapid and efficient manner but also improve blood circulation of the gastric mucosa [3–4]. However, PSS does not conform to normal physiology. It reduces the blood flow from the portal vein to the liver and even causes blood outflow from the liver, resulting in further liver dysfunction after operation [5]. The incidence of hepatic encephalopathy is high, and the operation is very traumatic. Devascularization is a surgical procedure to reduce or block the portal blood flow and the communicating veins of the portal vein and the azygos vein system. In 1929, Walter first reported ligation of the gastric coronary vein, following which several types of devascularization procedures were followed. Among them, splenectomy plus esophagogastric devascularization (EGDV) is the most effective [6]; hence, it has become the first treatment choice in portal hypertension complicated with thoracic esophageal varices and bleeding, in China. In terms of clinical research development, especially the anatomical study of the lower esophagus and cardia, selective esophagogastric devascularization (s-EGDV) has gained more popularity. s-EGDV is an improved surgical method based on traditional pericardial devascularization; s-EGDV only devascularizes the branch veins that enter the esophagus and stomach wall. Compared with traditional pericardial devascularization, s-EGDV not only devascularizes the branch veins to prevent bleeding but also retains the integrity of the gastric coronary vein and paraesophageal veins and maintains spontaneous portacaval shunt. The selective process guarantees spontaneous shunt of the body's part of the portal vein and ensures the direction of blood flow from the gastric coronary vein to the paraesophageal veins to the semi-azygos vein. Portoazygous spontaneously shunt, which forms a shunt between the portal and azygos vein, offers a compensatory mechanism, as the resulting blood flow is reasonable, appropriate, and physiologically compatible. It can maintain the necessary hepatic blood flow and appropriately reduce portal vein pressure to achieve a dynamic balance. It is different from the man-made spleen-renal shunt or portal vein-vena cava shunt, this spontaneously shunt should be retained. s-EGDV can preserve this spontaneous shunt on the basis of devascularization, which can achieve combined operation of the shunt and devascularization. Some randomized controlled trials (RCTs) and observational clinical studies (OCSs) were conducted to address this issue. Several published studies have shown convincing results in recent years. Therefore, this meta-analysis aimed to compare the perioperative outcomes and postoperative complications between s-EGDV and n-sEGDV.

## MATERIALS AND METHODS

### Literature search

We searched the databases of the Cochrane Library, PubMed, EMBASE, web of Science, TCGA and the Chinese Biomedicine Database from January 2000 to June 2017 both electronically and manually following search terms: ‘selective esophagogastric devascularization’ OR ‘s-EGDV’ OR ‘selective pericardial devascularization’ And ‘esophagogastric devascularization’ OR ‘EGDV’ OR ‘pericardial devascularization’ And ‘Non-Selective esophagogastric devascularization’ OR ‘n-sEGDV’ OR ‘conventional pericardial devascularization’ And ‘portal hypertension’. Both MeSH words and free terms were included in the search, No language restriction and two independent researchers performed this search. Final inclusion was determined by consensus. The results of the search strategy are shown in Table 1.

**Table 1:**
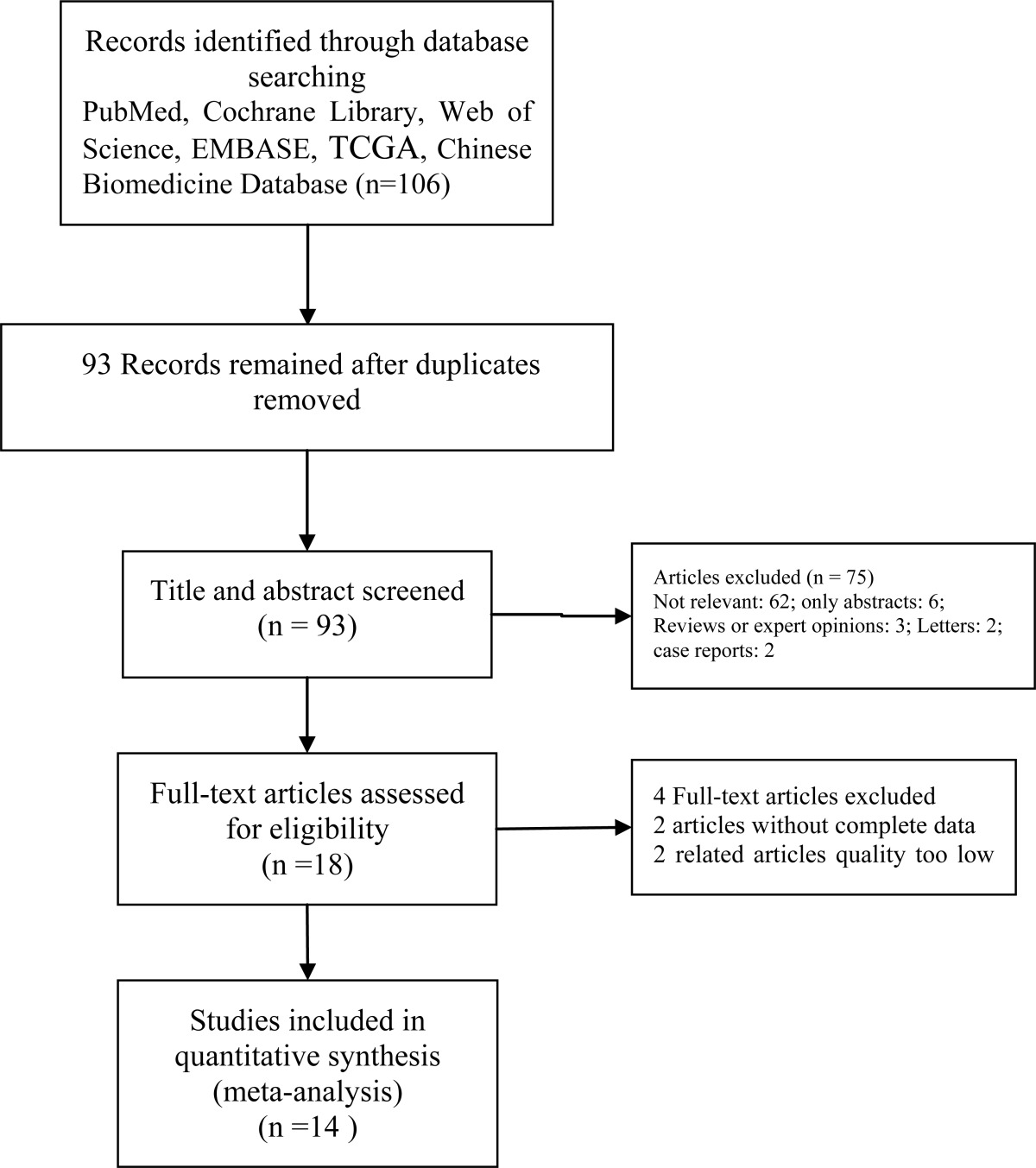
Flow diagram

### Inclusion criteria

The following studies were included: (1) those that compared the perioperative outcomes and postoperative complications between s-EGDV and n-sEGDV for cirrhotic patients with portal hypertension complicated with thoracic esophageal varices and bleeding; and (2) those that were the most recent publication in the case of multiple similar reports.

### Exclusion criteria

The following studies were excluded: (1) those wherein the detailed surgical type was not reported; (2) those with no comparison of s-EGDV and n-sEGDV; (3) those in which the study outcomes did not include complete or available perioperative outcomes and postoperative data; (4) those which reported data used in a later study; and (5) case reports, abstracts, letters, comments, reviews, guideline articles without original data, and studies that presented insufficient data.

### Data extraction

The following detailed data were extracted by two independent investigators: authors; year of publication; country; study design; surgery type; number of patients; and the following clinical data: (1) The decrement of FPP (cm H_2_O): select the right gastroepiploic vein catheter to portal vein, the zero point in axillary midline, the mean value of 3 time as the value of preoperative FPP, stop operation to complete 10 min measured again after 3 times, the average value as the FPP value after operation. The difference between the two is FPP reduction. (2) The value of PVF (mL/min): The portal vein diameter (D) and maximum flow velocity (Vmax) were measured by ultrasound before and three months after surgery. Then, the value of PVF was calculated according to the formula Q = Л/4D × 0.57 Vmax × 60. (3) Hepatic artery flow (HAF): The hepatic artery diameter (D) and maximum flow velocity (Vmax) were measured by ultrasound before and three months after surgery. Then, the value of HAF was calculated according to the formula Q = Л/4D × 0.57 Vmax × 60. (4) Portal hypertensive gastropathy (PHG), (5) hepatic encephalopathy, (6) postoperative re-bleeding rate; (7) postoperative mortality.

### Statistical analysis

Meta-analysis was conducted with Review Manager (version 5.3.0) software. Odds ratios (ORs) were used to analysis the dichotomous variables and 95% confidence interval (CI) values were reported. The Mantel-Haenszel, Chi-square, and I^2^ tests were used to test the heterogeneity between studies. *I*^2^ > 50%, this suggested significant heterogeneity, a random effects model was applied. If I^2^ < 50%, this suggested not significant heterogeneity, a fixed effects model was applied. If *P* < 0.05, this considered statistically significant. Funnel plots were used to evaluate potential publication bias.

### Characteristics of the included studies and quality assessment

14 studies (seven randomized clinical trials RCTs and seven retrospective cohort studies) were included in this meta-analysis. The total number of patients was 1637, of whom 766 was s-EGDV group and 871 was n-sEGDV group. The detailed characteristics of all the included studies are shown in Table 2.

**Table 2:** The characteristics of all the included studies

Author	year	Country	Study type	Group	Patients number	Male/female	Age, y	Study quality RCT (jadad system) retro (NOS system)
Zhao B *et al*. [7]	2016	China	RCT	s-EGDV	40	-	-	5
				n-sEGDV	40	-	-	
Zhang SJ *et al*. [8]	2014	China	RCT	s-EGDV	58	32/26	48.12 ± 9.34	7
				n-sEGDV	58	31/27	49.08 ± 9.21	
Wang C *et al*. [9]	2014	China	RCT	s-EGDV	90	40/50	47 ± 13	7
				n-sEGDV	90	45/45	43 ± 11	
Gu GJ *et al*. [10]	2013	China	RCT	s-EGDV	30	21/9	45.7 ± 4.7	5
				n-sEGDV	30	23/7	45.9 ± 4.5	
Gong QH *et al*. [11]	2013	China	RCT	s-EGDV	93	67/26	41.3 ± 7.5	7
				n-sEGDV	93	68/25	41.7 ± 6.9	
Wang WS *et al*. [12]	2012	China	RCT	s-EGDV	52	-	-	7
				n-sEGDV	50	-	-	
Pan WN *et al*. [13]	2009	China	RCT	s-EGDV	91	59/32	47 (32–69)	7
				n-sEGDV	85	55/30	48 (27–71)	
Zou SH *et al*. [14]	2017	China	OCS	s-EGDV	32			5
				n-sEGDV	30			
Zhang Y *et al*. [15]	2016	China	OCS	s-EGDV	55	34/21	45 ± 17	5
				n-sEGDV	89	49/40	43 ±1 5	
Liu GF *et al*. [16]	2013	China	OCS	s-EGDV	48	28/20	58.26 ± 10.29	7
				n-sEGDV	48	27/21	57.75 ± 11.16	
Ren DF *et al*. [17]	2013	China	OCS	s-EGDV	45	33/12	42	5
				n-sEGDV	41	30/11	45	
You DY *et al*. [18]	2013	China	OCS	s-EGDV	28	-	-	5
				n-sEGDV	62	-	-	
Zhao H *et al*. [19]	2011	China	OCS	s-EGDV	57	35/22	42 ± 16	5
				n-sEGDV	51	30/21	43 ± 12	
Cen J *et al*. [20]	2005	China	OCS	s-EGDV	47	35/12	44.6 (21–72)	7
				n-sEGDV	104	76/28	43.5 (26–67)	

RCT = randomized controlled trial; OCS: retrospective cohort studies. Jadad scale system: The Jadad scale, sometimes known as Jadad scoring or the Oxford quality scoring system, is a procedure to independently assess the methodological quality of a clinical trial. It is the most widely used such assessment in the world; The system was used to assess randomization, concealment of allocation, blinding, and withdrawals in the study. Each item was given a score of 0–2 and 7 score in total. If the total score was ≥4, the RCT was of high quality. The Newcastle-Ottawa System: The quality of the nonrandomized studies was assessed by using this System, The quality of the studies was evaluated by examining three items: patient selection, comparability of groups, and assessment of outcome. Each study was given 9 score in total; if the total score was ≥7, the OCS was considered to be of high quality

### Assessment of the risk of bias of RCTs

**Figure 1:**
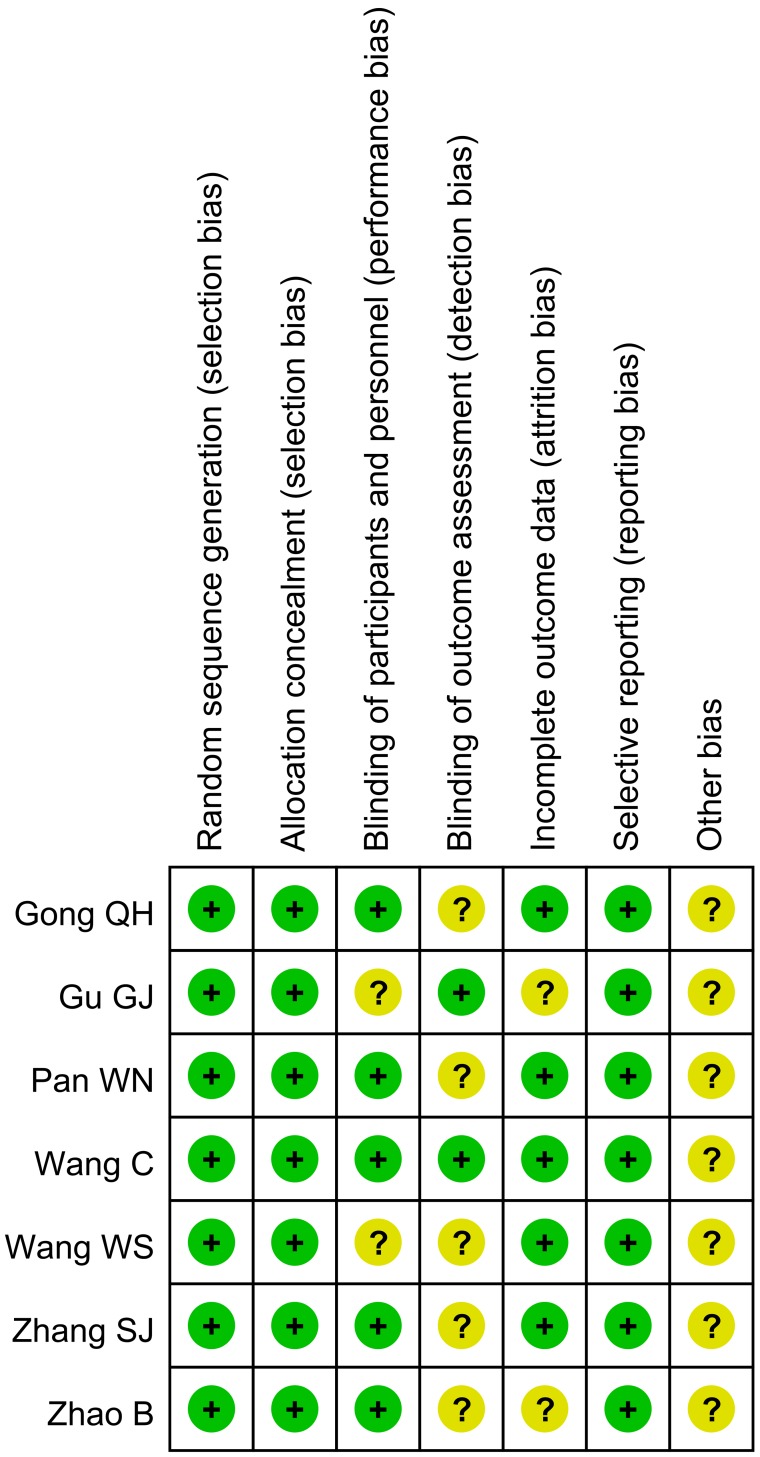
Risk of bias of RCTs: Assessment was based on a quality checklist recommended in the Cochrane Handbook “Yes” indicated a “low” risk of bias; “unclear,” an “uncertain” risk of bias; “no,” a “high” risk of bias.

For the included RCTs, assessment of the bias risk involved six parameters: allocation concealment, incomplete outcome data, blinding, selective reporting bias, sequence generation, and other potential sources of bias. Assessment was based on a quality checklist recommended in the Cochrane Handbook. “Yes” indicated a “low” risk of bias; “unclear,” an “uncertain” risk of bias; “no,” a “high” risk of bias (Figure 1).

## META-ANALYSIS RESULTS

### The reduction of PVF

**Figure 2:**
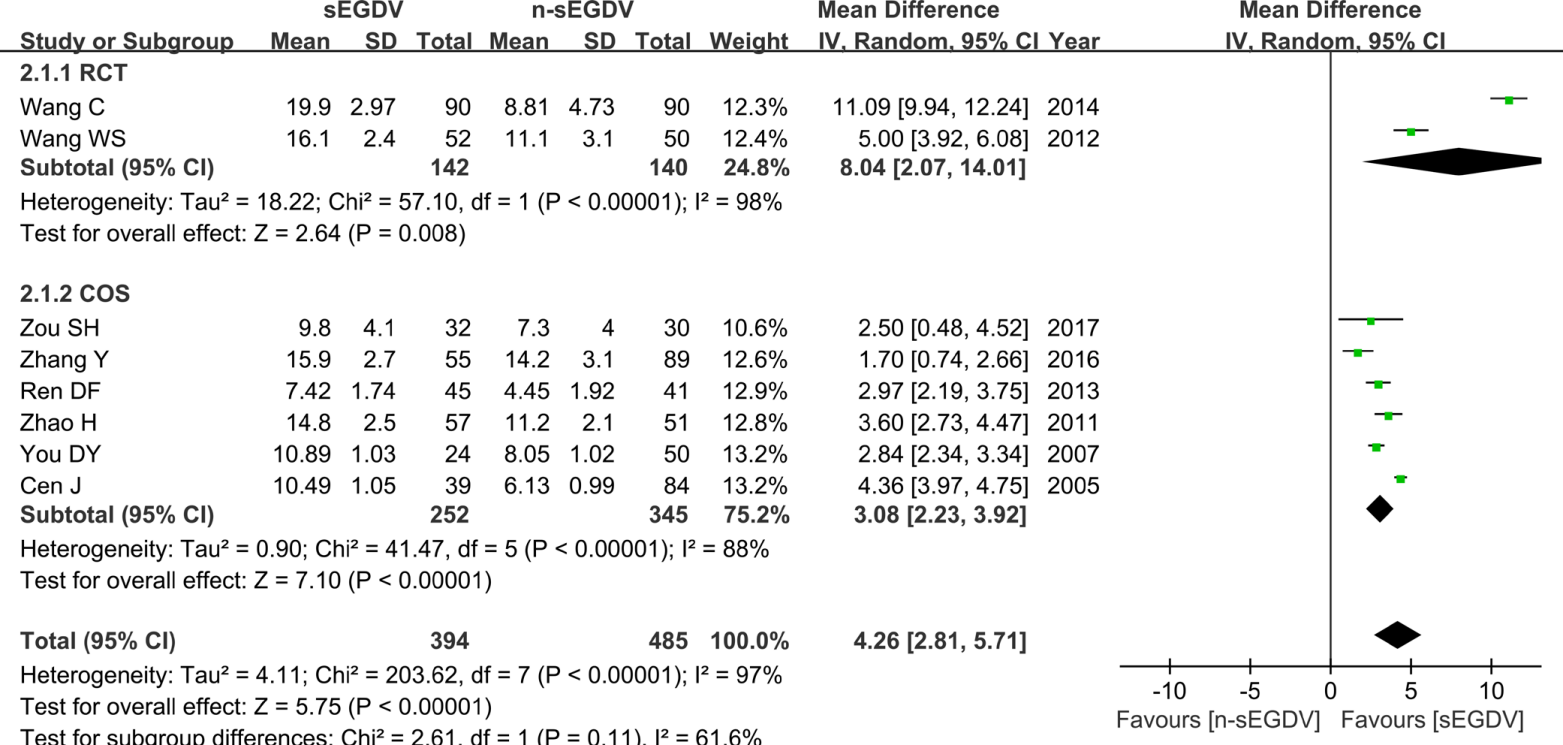
Meta-analysis of the reduction of PVF

Eight included studies reported the reduction of PVF, we pooled data from the eight studies to comparing n-sEGDV group with sEGDV group. The results of meta-analysis indicate that there is significant difference between two groups in the reduction of PVF (OR = 4.26; 95% CI, 2.81–5.71; *P* < 0.00001; I^2^ = 97% for heterogeneity), Therefore, using a Random model. The meta-analysis of RCTs and OCS subgroup both reveals a statically different between two groups in the reduction of PVF. [RCTs (I^2^ = 98%, OR = 8.04; 95% CI, 2.07–14.01; *P* = 0.008), OCS (I^2^ = 88%, OR = 3.08; 95% CI, 2.23–3.92; *P* < 0.00001)] (Figure 2).

### Portal vein flow

**Figure 3:**
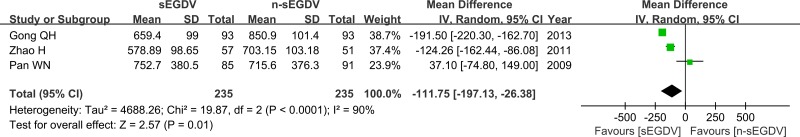
Meta-analysis of portal vein flow

Three included studies reported the portal vein flow. The results of meta-analysis show that there is no difference between two groups in the portal vein flow (OR = −111.75; 95% CI, −197.13–26.38; *P* = 0.01; *I*^2^ = 90% for heterogeneity), Therefore, using a Random model (Figure 3).

### Hepatic artery flow

**Figure 4:**
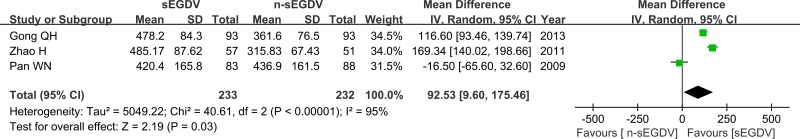
Meta-analysis of hepatic artery flow

Three included studies reported the hepatic artery flow. The results of meta-analysis show that there is no difference between two groups in the hepatic artery flow (OR = 92.53; 95% CI, 9.60–175.46; *P* = 0.03; I^2^ = 95% for heterogeneity), Therefore, using a Random model (Figure 4).

### Portal hypertensive gastropathy

**Figure 5:**
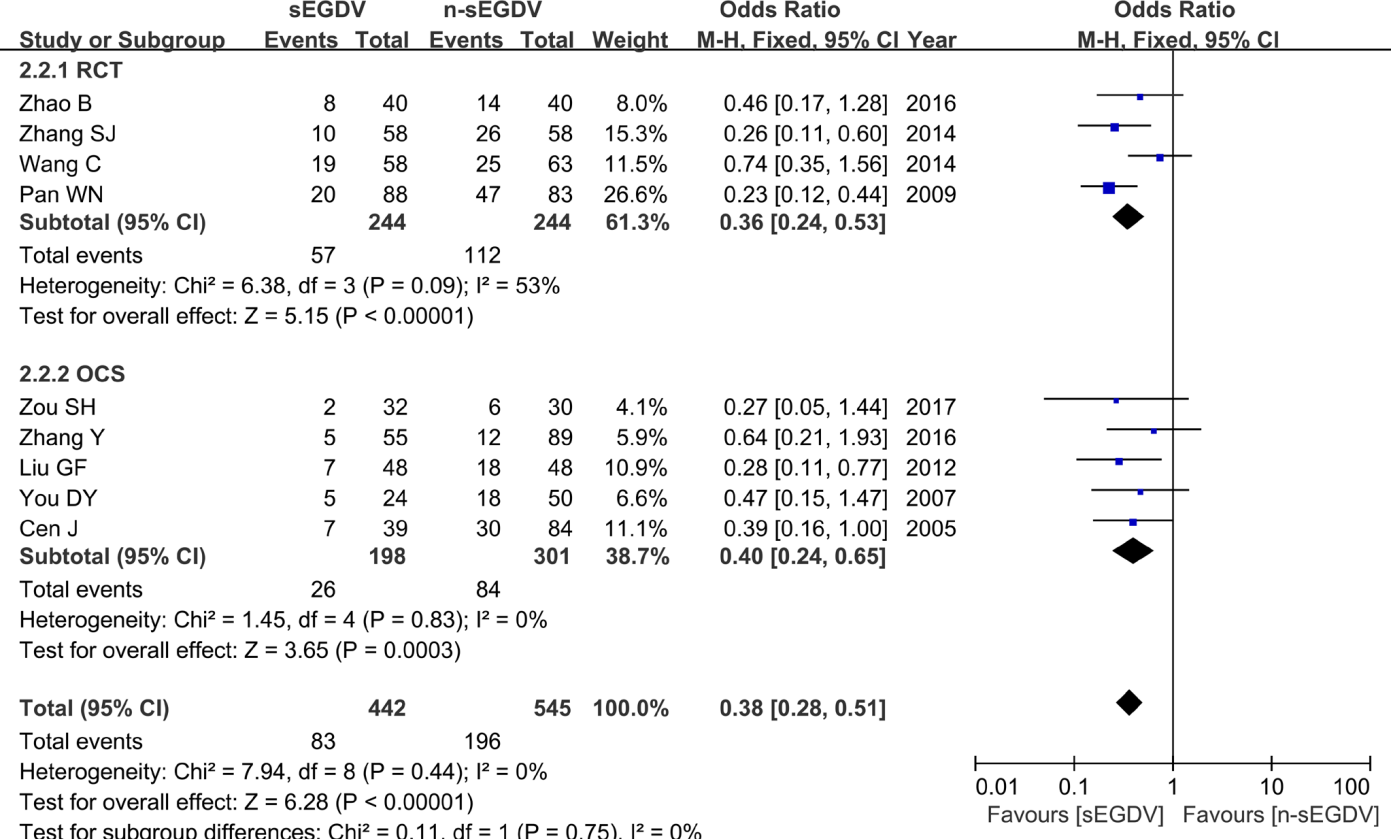
Meta-analysis of portal hypertensive gastropathy

Nine included studies reported the portal hypertensive gastropathy. The results of meta-analysis show that there is difference between two groups in the portal hypertensive gastropathy (OR = 0.38; 95% CI, 0.28–0.51; *P <* 0.00001; *I*^2^ = 0% for heterogeneity), Therefore, using a fixed model. The meta-analysis of RCTs and OCS subgroup both reveals a statically different between two groups in the portal hypertensive gastropathy. [RCTs (*I*^2^ = 53%, OR = 0.36; 95% CI, 0.24–0.53; *P <* 0.00001), OCS (I^2^ = 0%, OR = 0.40; 95% CI, 0.24–0.65; *P* = 0.0003)] (Figure 5).

### Hepatic encephalopathy

**Figure 6:**
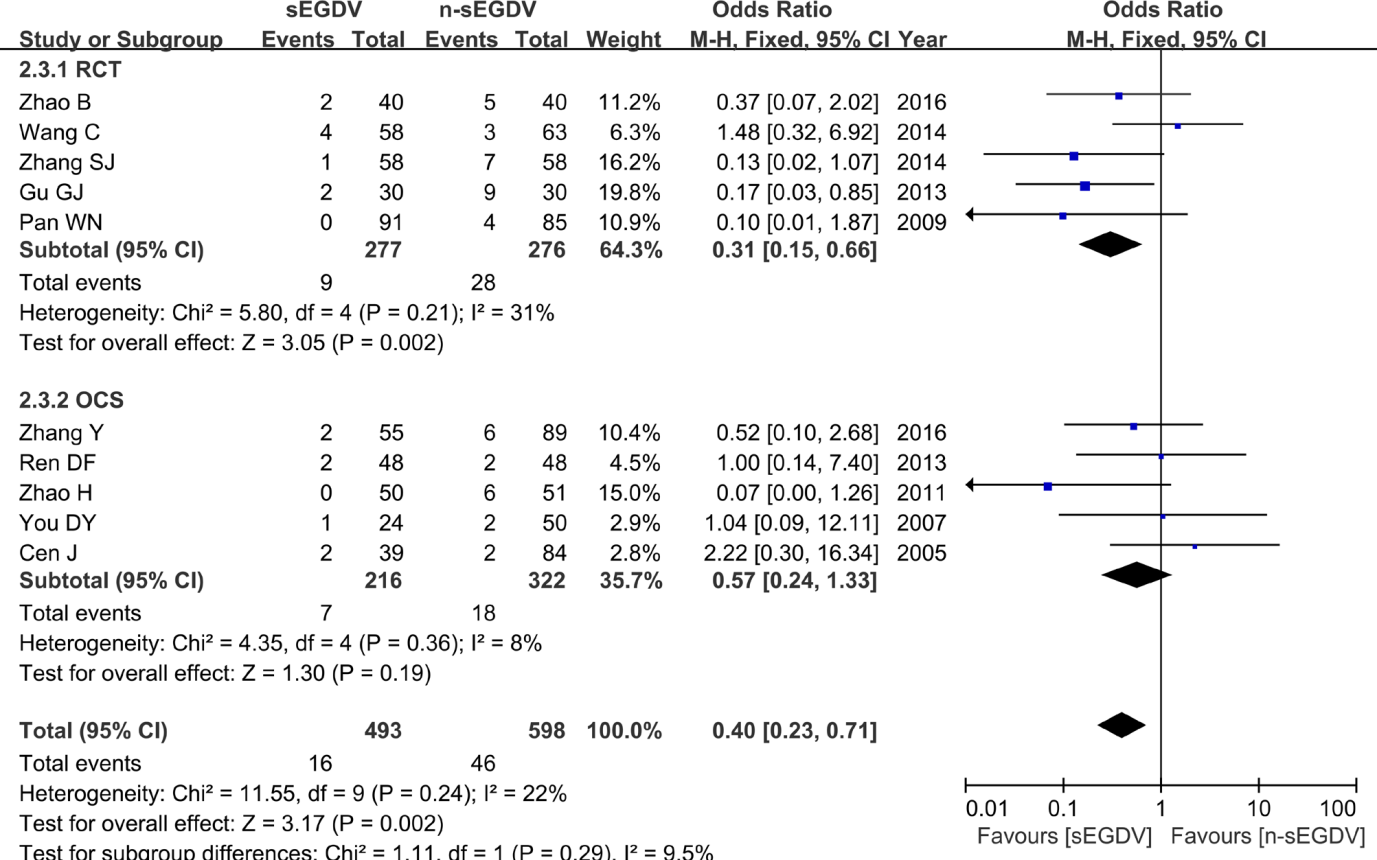
Meta-analysis of hepatic encephalopathy

Ten included studies reported the incidence of hepatic encephalopathy. The results of meta-analysis show that there is no significant difference between two groups in the incidence of hepatic encephalopathy (OR = 0.40; 95% CI, 0.23–0.71; *P* = 0.002; *I*^2^ = 22% for heterogeneity), Therefore, using a fixed model. The meta-analysis of RCTs and OCS subgroup both reveals a statically different between two groups in the incidence of hepatic encephalopathy. [RCTs (*I*^2^ = 31%, OR = 0.31; 95% CI, 0.15–0.66; *P* = 0.002), OCS (*I*^2^ = 8%, OR = 0.57; 95% CI, 0.24–1.33; *P* = 0.019)] (Figure 6).

### Postoperative re-bleeding

**Figure 7:**
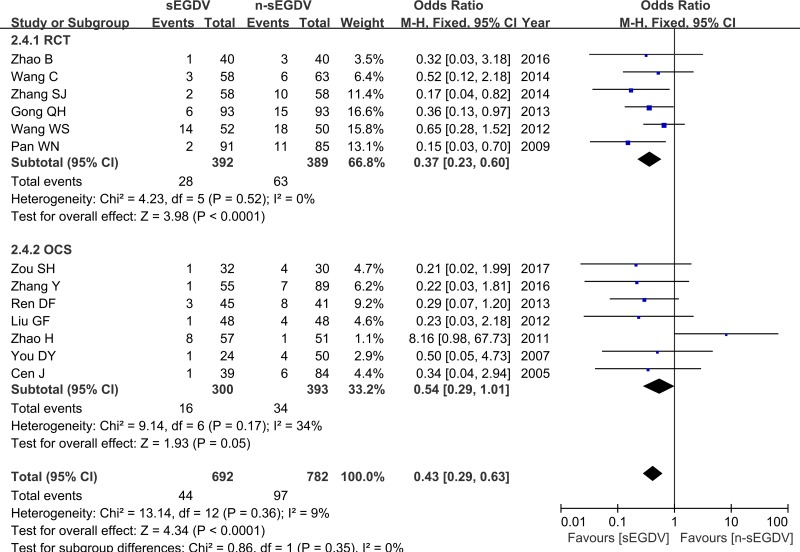
Meta-analysis of postoperative re-bleeding

Thirteen included studies reported the incidence of postoperative re-bleeding. The results of meta-analysis show that there is significant difference between two groups in the incidence of postoperative re-bleeding (OR = 0.43; 95% CI, 0.29–0.63; *P <* 0.0001; *I*^2^ = 9% for heterogeneity), Therefore, using a fixed model. The meta-analysis of RCTs and OCS subgroup both reveals a statically different between two groups in the incidence of postoperative re-bleeding. [RCTs (*I*^2^ = 0%, OR = 0.37; 95% CI, 0.23–0.60; *P <* 0.0001), OCS (*I*^2^ = 34%, OR = 0.17; 95% CI, 0.29–1.01; *P* = 0.05)] (Figure 7).

### Postoperative mortality

**Figure 8:**
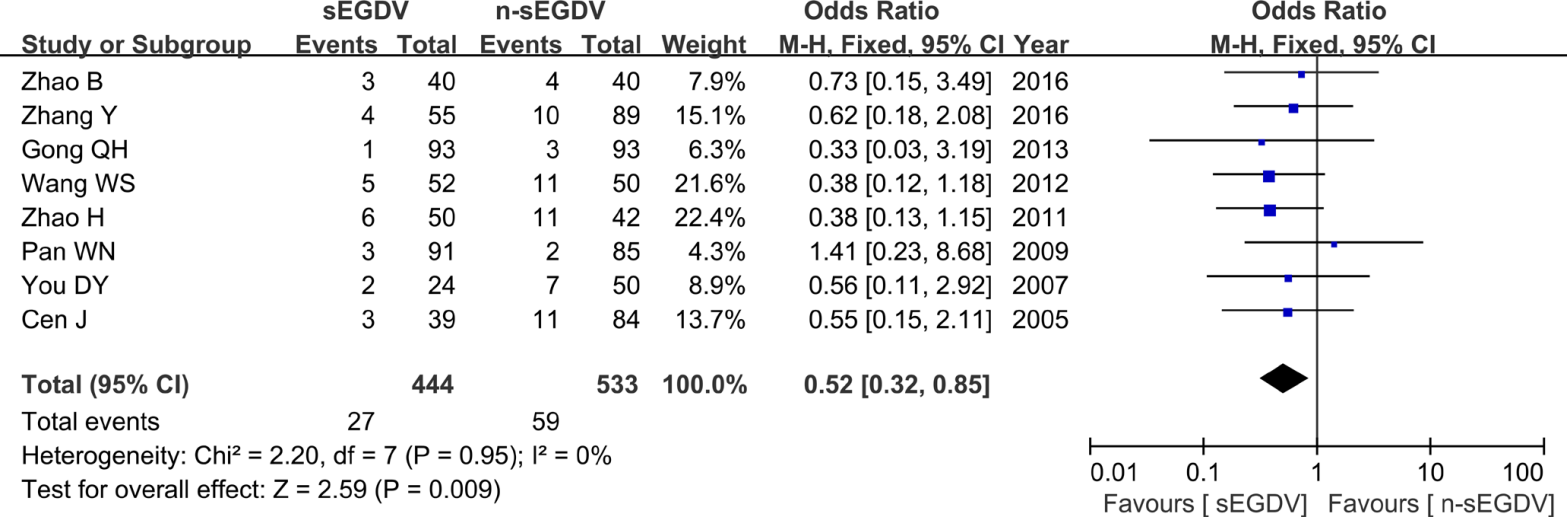
Meta-analysis of postoperative mortality

Eight included studies reported the postoperative mortality three year after operation. The results of meta-analysis show that there is significant difference between two groups in the incidence of postoperative mortality (OR = 0.52; 95% CI, 0.32–0.85; *P* = 0.009; I^2^ = 0% for heterogeneity), Therefore, using a Fixed model (Figure 8).

### Publication bias

**Figure 9:**
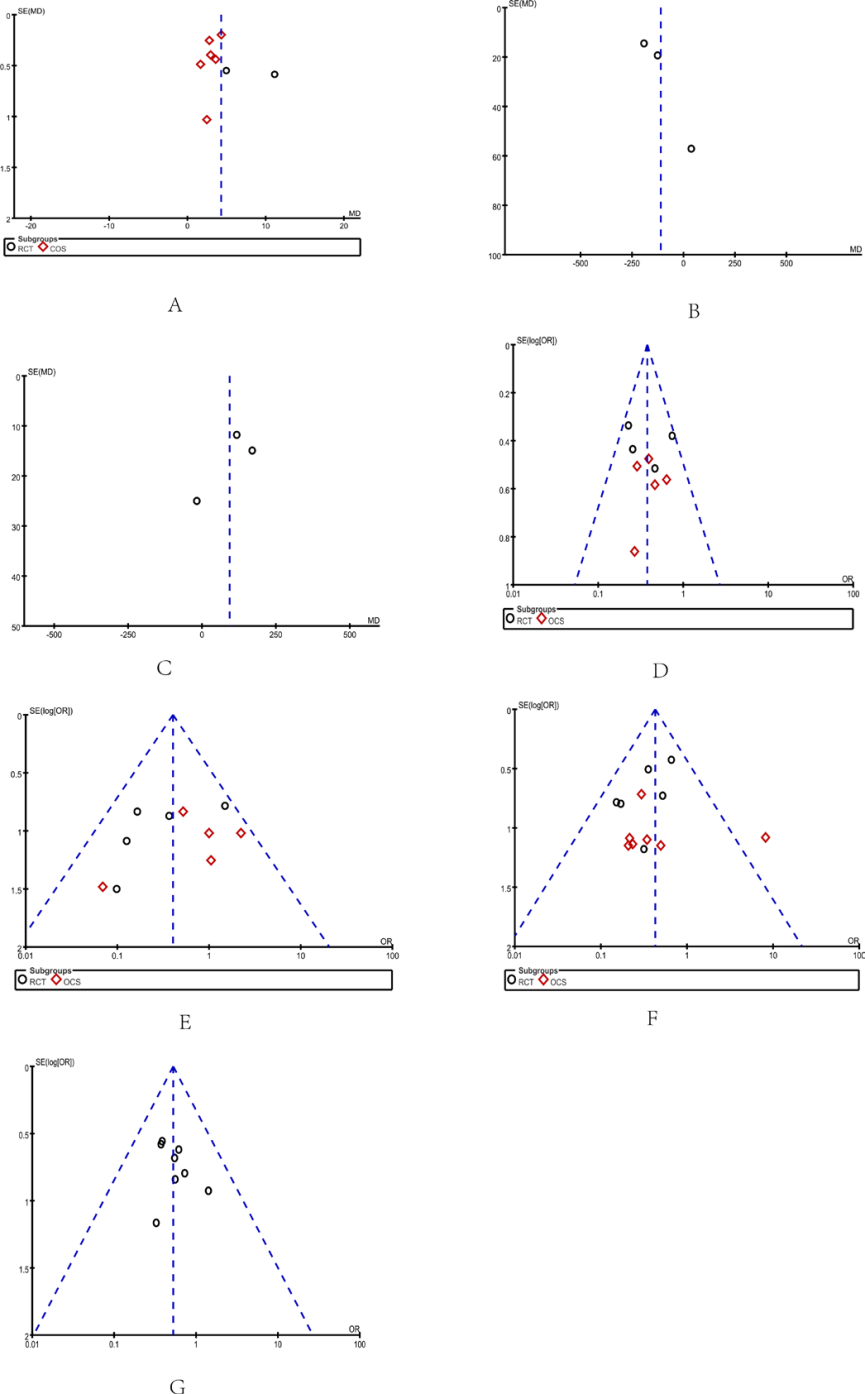
Funnel plots: Funnel plots were created to assess the publication bias in our meta-analysis of included studies In the absence of publication bias, it assumes that studies with high precision will be plotted near the average, and studies with low precision will be spread evenly on both sides of the average, creating a roughly funnel-shaped distribution. (**A**) Reduction of PVF (**B**) Portal vein flow (**C**) Hepatic artery flow (**D**) Portal hypertensive gastropathy (**E**) Hepatic encephalopathy (**F**) Postoperative re-bleeding (**G**) Postoperative mortality

Deviation from this shape in Funnel plots can indicate publication bias. There was no evident asymmetry in the funnel plots (Figure 9), suggesting a low probability of publication bias.

## DISCUSSION

Surgical treatment for liver cirrhosis complicated with portal hypertension mainly focuses on the prevention and treatment of esophageal varices bleeding, elimination of ascites, reduction of complications, and improvement in the overall quality of life [21]. An important factor to consider for evaluation of surgical approach for the treatment of portal hypertension is portal blood flow reduction as well as maintenance of hepatic blood inflow to prevent liver dysfunction and hepatic encephalopathy. There was a statistically significant difference in the PVF reduction between the two groups analyzed in this report. The results of our meta-analysis show that sEGDV was superior to n-sEGDV in reduction of PVF. Confirmed the effect of spontaneous portacaval shunt in decrease the PVF.

In recent years, many studies have shown that portal vein blood flow is reduced after devascularization, and that hepatic arterial blood flow increases as compensation to maintain the necessary blood supply to the liver [22]. In this study, the portal venous blood flow was reduced in both the sEGDV group and n-sEGDV group, and the postoperative blood flow of the portal vein in the sEGDV group was significantly lower than that in the n-sEGDV group. The postoperative arterial blood flow in the sEGDV and n-sEGDV groups all showed compensatory increase, and our meta-analysis results showed that the postoperative arterial blood flow of the portal vein in the sEGDV group was significantly higher than that in the n-sEGDV group. The mechanism is sEGDV group retained spontaneous shunt and portal vein blood flow decreased significantly, so the portal venous pressure and liver sinus pressure decreased significantly in the sEGDV group than in the n-sEGDV group. The hepatic artery velocity and flow showed an increase, while nitric oxide synthesis was inactivated and other vasodilator substances in the liver decreased due to the spontaneous shunt. These vasodilator substances resulted in the increase of hepatic artery blood flow and the ratio of hepatic artery in liver blood supply.

sEGDV preserves the continuous of portoazygous spontaneously shunt can alleviate visceral congestion and reduce the incidence of portal vein thrombosis and portal hypertensive gastropathy, thereby increasing the incidence of postoperative portal hypertension gastropathy [23–25]. This meta-analysis also confirmed that sEGDV was superior to n-sEGDV in reducing the rate of postoperative portal hypertension gastropathy. Compared to PSS, esophagogastric devascularization had no significant effect on the blood flow and function of the liver, which can prevent hepatic encephalopathy after operation. Our study showed that no significant difference existed between the sEGDV and n-sEGDV groups with respect to the rate of postoperative hepatic encephalopathy.

The reduction in the rate of re-bleeding resulting from moderation of hyperpressure of the portal vein should also be considered. Splenectomy significantly reduces the blood flow of the portal vein and decreases portal venous pressure. The traditional esophagogastric devascularization completely blocks the area around the esophagus and gastric fundus blood vessels and promotes the formation of new traffic branch veins of the portal vein and the azygos venous system, eventually leading to recurrence of esophageal varices and bleeding. Selective devascularization avoids the complete disconnection of shunt vessels and preserves the continuity of the portoazygous spontaneously shunt. Therefore, sEGDV can decrease the recurrence rate of varicose veins and rate of re-bleeding. The result of our meta-analysis also confirmed that sEGDV was superior to n-sEGDV in reducing the rate of postoperative re-bleeding.

The postoperative mortality was significantly lower in the sEGDV group than in the n-sEGDV group [26–27]. This may be due to lower incidence of re-bleeding and hepatic encephalopathy in the sEGDV group. Thus, sEGDV was a more effective treatment with fewer complications for portal hypertension complicated with thoracic esophageal varices and bleeding than n-sEGDV. However, the paraesophageal veins cannot be preserved in every patient. If the vein trunk of the paraesophageal veins directly enter the esophageal wall, or it is difficult to identify the paraesophageal vein due to the varicose vessels from the venous plexus or bolus, then it should be devascularized from the beginning of the gastric coronary vein to ensure that the abnormal blood flow to the esophagogastric varices is blocked, to control and reduce the fatal variceal bleeding.

A major limitation of our meta-analysis is that it only included a small number of high-quality RCTs, all coming from the same country—China. Another potential limitation is that surgical experience and methods used at different hospitals and specialist centers could have produced different outcomes and increased the heterogeneity between the included studies. In addition, the treatment of complications may have affected the outcome of the RCTs and OCSs included in this meta-analysis.

## CONCLUSIONS

sEGDV offers a more effective surgical approach with fewer complications to treat portal hypertension than n-sEGDV. Upon further detailed analysis of the surgical indications and hemodynamic and postoperative major complications of selective devascularization, sEGDV likely will provide us with a new direction in the choice of surgical approach for portal hypertension.
